# Genomic Instability-Related LncRNA Signature Predicts the Prognosis and Highlights *LINC01614* Is a Tumor Microenvironment-Related Oncogenic lncRNA of Papillary Thyroid Carcinoma

**DOI:** 10.3389/fonc.2021.737867

**Published:** 2021-09-16

**Authors:** Xubin Dong, Cong Jin, Danxiang Chen, Yizuo Chen, Zhi-qiang Ye, Xiaohua Zhang, Xiaoli Huang, Wei Zhang, Dian-na Gu

**Affiliations:** ^1^Department of Breast Surgery, The First Affiliated Hospital of Wenzhou Medical University, Wenzhou, China; ^2^Department of Thyroid Surgery, The First Affiliated Hospital of Wenzhou Medical University, Wenzhou, China; ^3^Department of Oncology, The First Affiliated Hospital of Wenzhou Medical University, Wenzhou, China

**Keywords:** genomic instability (GI), mutator phenotype, long non-coding RNAs, papillary thyroid carcinoma, prognosis

## Abstract

**Background:**

Genomic instability (GI) is among the top ten characteristics of malignancy. Long non-coding RNAs (lncRNAs) are promising cancer biomarkers that are reportedly involved in GI. So far, the clinical value of GI-related lncRNAs (GIlncs) in papillary thyroid cancer (PTC) has not been clarified.

**Methods:**

Integrative analysis of lncRNA expression and somatic mutation profiles was performed to identify GIlncs. Analysis of differentially expressed lncRNAs in the group with high- and low- cumulative number of somatic mutations revealed significant GIlncs in PTC. Univariate and multivariate Cox proportional hazard regression analyses were performed to identify hub-GIlncs.

**Results:**

A computational model based on four lncRNAs (*FOXD2-AS1*, *LINC01614*, *AC073257.2*, and *AC005082.1*) was identified as a quantitative index using an *in-silicon* discovery cohort. GILS score was significantly associated with poor prognosis, as validated in the TCGA dataset and further tested in our local RNA-Seq cohort. Moreover, a combination of clinical characteristics and the composite GILS-clinical prognostic nomogram demonstrates satisfactory discrimination and calibration. Furthermore, the GILS score and *FOXD2-AS1*, *LINC01614*, *AC073257.2*, and *AC005082.1* were also associated with driver mutations and multiple clinical-pathological variables, respectively. Moreover, RNA-Seq confirmed the expression patterns of *FOXD2-AS1*, *LINC01614*, *AC073257.2*, and *AC005082.1* in PTC and normal thyroid tissues. Biological experiments demonstrated that downregulated or overexpressed *LINC01614* affect PTC cell proliferation, migration, and invasion *in vitro*. Activation of the stromal and immune cell infiltration was also observed in the high *LINC01614* group in the PTC microenvironment.

**Conclusion:**

In summary, we identified a signature for clinical outcome prediction in PTC comprising four lncRNAs associated with GI. A better understanding of the GI providing an alternative evaluation of the progression risk of PTC. Our study also demonstrated *LINC01614* as a novel oncogenic lncRNA and verified its phenotype in PTC.

## Introduction

Papillary thyroid cancer (PTC) is the most common type of thyroid cancer, with heterogeneous biological behavior and a favorable prognosis (1–3). In a retrospective study of PTC with 27 years of median follow-up times, the recurrence rate and PTC-specific death rate were 28% and 9%, respectively (4). Although the overall prognosis of most PTC patients was satisfactory, there was still a small part of PTC patients having aggressive characteristics, and even after standard surgical treatment, they are still prone to recurrence and metastasis (5).

Genomic instability (GI) endowed tumors with an inherent survival advantage. As a hallmark of cancer, GI triggers self-sufficiency in evasion of programmed cell death, limitless replicative potential, sustained angiogenesis, and tissue invasion (6, 7). PTC exhibits frequent alterations in oncogenes (such as *BRAF* and *RAS*), DNA repair defects, and GI (8). Emerging studies demonstrated that GI was highly regulated through DNA damage checkpoints, DNA repair mechanisms, and mitotic checkpoints. Besides, aberrant transcriptional regulation and epigenetic modification are also implicated in genome instability. The exploration of a novel biomarker utilizing the GI signature of PTC might be helpful for risk stratification and prognostic evaluation of PTC patients.

Increasing evidence has shown that many epigenetic regulators in tumors reside in non-coding regions, mostly transcribed into long non-coding RNAs (lncRNAs), which transcripts of more than 200 nucleotides, involved in the survival, proliferation, migration, and genomic stability of cells (9, 10). LncRNA *NORAD*, *CUPID1*, *CUPID2*, and *DDSR1* promote genomic stability by regulating the expression of DNA repair-associated genes or by interacting with damage-related proteins (11–13). A recent study revealed that *BGL3* lncRNA mediates BRCA1/BARD1 retention at double-strand breaks (14). lncRNAs act as vital epigenetic regulators of multiple biological processes by binding to different DNA, mRNA, or proteins and are requisite in regulating and maintaining genomic stability and tumorigenesis (15). These mechanisms by which lncRNAs regulate DNA repair-associated genes facilitate our understanding of the link between lncRNA and GI. Moreover, several lncRNA signatures have been established for predicting the prognosis of cancer patients (16–20), whereas the potential biological and clinical significance of GI-associated lncRNAs in PTC remains largely unknown.

In the following research, we constructed a lncRNA signature from the genomic and transcriptional levels to predict the clinical outcome of PTC. Besides, we also investigated the molecular changes related to PTC associated with the GILS score. GILS predicted the function of *LINC01614* in mediating cell proliferation and migration, which was further validated by overexpression and knockdown experiments in PTC cell lines. In summary, our findings showed that the GILS robustly predict patient prognosis and revealed the oncogenic functions of *LINC01614*, which have great potentials in the future development of PTC biomarkers.

## Materials and Methods

### Public Dataset Source and Preprocessing

A total of 568 thyroid cancer RNA-Seq profile samples in TCGA, including 58 matched normal samples, 502 thyroid cancer samples, and eight metastatic thyroid carcinoma samples, were downloaded using the “TCGAbiolinks” package (21). Whole-transcriptome sequencing data was performed using FPKM expression level in transcripts per million (TPM). The definition and outcome of progression-free survival (PFS) were obtained from the TCGA-Clinical Data Resource (CDR) (22). Mutation status was obtained from Mutation Annotation Format files (derived from MuTect2) from the Genomic Data Commons portal. After excluding two patients with follicular thyroid cancer and two patients who received pretreatment, the expression data, prognostic information, and mutation profiles were included in 487 patients in this study. To exclude genes with high variability across patients, we calculated the median absolute deviation (MAD) of the 487 samples. LncRNAs with MAD > 0.5 were defined as genes with high variability and excluded in the RNA-Seq matrix.

### Clinical Specimens and RNA Sequencing

Seventy-nine pairs of thyroid tissues were obtained from the Department of Thyroid Surgery, The First Affiliated Hospital of Wenzhou Medical University. Fresh tissues were immediately snap-frozen in liquid nitrogen and stored at -80°C until further use. All pathological reports were independently confirmed by two experienced pathologists.

The RNA-seq experimental protocol was performed as described in a previous publication (23). Briefly, total RNA was used to construct cDNA libraries through high-throughput RNA sequencing. The RNA expression profile was determined from the sequencing libraries generated from a NEBNext Ultra RNA Library Prep Kit for Illumina (NEB, United States). The clustering of samples was performed on a cBot Cluster Generation System using TruSeq PE Cluster Kit v3-cBot-HS (Illumina), and the library was sequenced on an Illumina NovaSeq platform. The read counts were normalized to TPM, and the TPM expression values were further log2 transformed.

### Establishment of GILS

To identify GIlncs, we examined differentially expressed genes (DEGs) in PTC from TCGA by Wilcoxon rank-sum test in “limma” package (|log_2_foldchange| > 0.5, false discovery rate (FDR) < 0.05). To assess the genomic instability, we proposed a mutator hypothesis-derived calculation method: we determined the cumulative number of somatic mutations based on the number of changed sites for each gene on each sample and categorized the patients in descending order. The top 20% of patients were titled with genomic unstable-like (GU-like) group and the last 20% as genomic stable-like (GS-like) group. Then, the GILS for prognosis prediction was developed based on the coefficient of each prognostic GIlncs in the model and their expression levels.

To evaluate the performance of GIlncs on the prognosis, GIlncs were selected by Univariate and multivariate Cox proportional hazard regression analysis. GIlncs with *p* < 0.05 in univariate Cox were retained, and multivariate Cox was performed in the model group by the “glmnet” package. Based on the coefficients from the multivariate regression analysis and the expression levels of prognostic GIlncs, we constructed a GI-associated lncRNA signature (GILS) for prognostic prediction as follows:


$$\text{GILS score}=\sum_{i=1}^{N}\text{Coefficient }{\left(\text{lncRNA}\right)}_{i}*\text{expression }{\left(\text{lncRNA}\right)}_{i}$$

Where *N* is the number of prognostic GILS, expression (lncRNA)*_i_* is the expression value of prognostic GILS*_i_*, and Coefficient (lncRNA)*_i_* is the estimated multivariable Cox regression coefficient of GILS*_i_*.

### Functional Enrichment Analysis

Function “cor. test” in R was used to measure the Spearman correlation coefficients between GI-lncRNAs and mRNAs, and the top 10 mRNAs were selected as PTC-specific lncRNA targets. Gene Ontology (GO) and Kyoto Encyclopedia of Genes and Genomes (KEGG) analysis were performed using “enrichGo” and “enrichKEGG” functions in the “clusterprofiler” package, respectively.

### TME Analysis

ESTIMATE is a ssGSEA based algorism for tumor-stroma purity detection, which uses the gene expression profiles of 141 immune and stromal genes. ESTIMATE score is a combination of immune and stromal scores, calculated by the “ESTIMATE” R package. The xCell project (24) used six public cells sorted bulk gene expression data sets to generate gene signatures and score each TCGA sample.

### RNA Extraction, and qRT-PCR

Total RNA was extracted using TRIzol Reagent (Invitrogen). Complementary DNA (cDNA) was synthesized using ReverTra Ace qPCR RT Kit (Toyobo, Japan). Real-time qPCR was performed using SYBR Select Master Mix on 7500 Fast Instrument. Each sample was tested in triplicate. Experiments were repeated three times. Human primer sequences for qPCR are as below: LINC01614, forward 5’-TCAACCAAGAGCGAAGCCAA-3’, reverse- 5'-TTGGACACAGACCCTAGCAC-3'; GAPDH, forward 5’-GTCTCCTCTGACTTCAACAGCG-3’, reverse- 5'-ACCACCCTGTTGCTGTAGCCAA-3’.

### Ectopic Expression and Gene Knockdown by siRNA

Full-length *LINC01614* cDNA was synthesized and inserted into pCDH-GFP+PURO-3xFlag and pCDH-GFP+PURO vectors (Genepharma, Shanghai, China). The resulting vector or empty vector was transfected into PTC cells using Lipofectamine 2000 Transfection Reagent (Life Technologies, Carlsbad, CA) according to the manufacturer’s protocol. Infected cells were selected with puromycin (Invivogen) at 1 μg/ml. For *LINC01614* knockdown, siRNA transfections were carried out using Lipofectamine RNAiMAX Reagent (Invitrogen). The siRNAs containing the following two individual siRNAs were used: si*LINC01614*#1: forward 5’-GCCCACCTCAAATCCTGAA-3’; si*LINC01614*#2: forward 5’-GCUGGAAGCAUUUCGUAAU-3’.

### Cellular Proliferation, Colony Formation, Migration, and Invasion Assay

After siRNA transfection, cells were trypsinized, resuspended, seeded in a 96-well plate with a density of 1.5×10^3^ cells/well, and incubated at 37°C. At each indicated time-point, 10 µl of CCK-8 was added and incubation was continued for 2 h. The plates were agitated and the absorbance was measured at 450 nm under an absorption spectrophotometer. For colony formation assay, cells were seeded in 6-well plates (1×10^3^ cells/well) and cultured at 37°C in 5% CO_2_. After 10 days, the cells were washed with PBS and stained with crystal violet. For migration assay, Cells were plated at a known density in the upper chamber of 8.0μM membrane transwells (Cat. #3422, Corning, Tewksbury, MA) in media containing 10% FBS. Transwells were placed in wells with media containing 10% FBS. Cells were fixed onto the transwell membrane in 10% formalin. Migrated or invaded cells were imaged in a 10× magnification microscope in 5-10 random fields for each well and quantified by ImageJ software.

### Statistical Analysis

Mann–Whitney test or Wilcoxon signed-rank test were used in the two-group analysis. Comparisons between different groups were conducted by Kruskal–Wallis or one-way ANOVA. Survival analysis was conducted using the Kaplan–Meier method and the log-rank test. Univariate and multivariate analyses with Cox proportional hazards regression for PFS were performed on the individual variables by calculating the hazard ratios (HR) and 95% confidence intervals (CI). The time-dependent ROC curve was calculated with the nearest neighbor estimation method. In all experiments, three biological replicates were performed for each group. All statistical analyses were performed using the R software (3.5.2).

## Results

### Identification of GIlncs in Papillary Thyroid Cancer Patients

**Figure 1:**
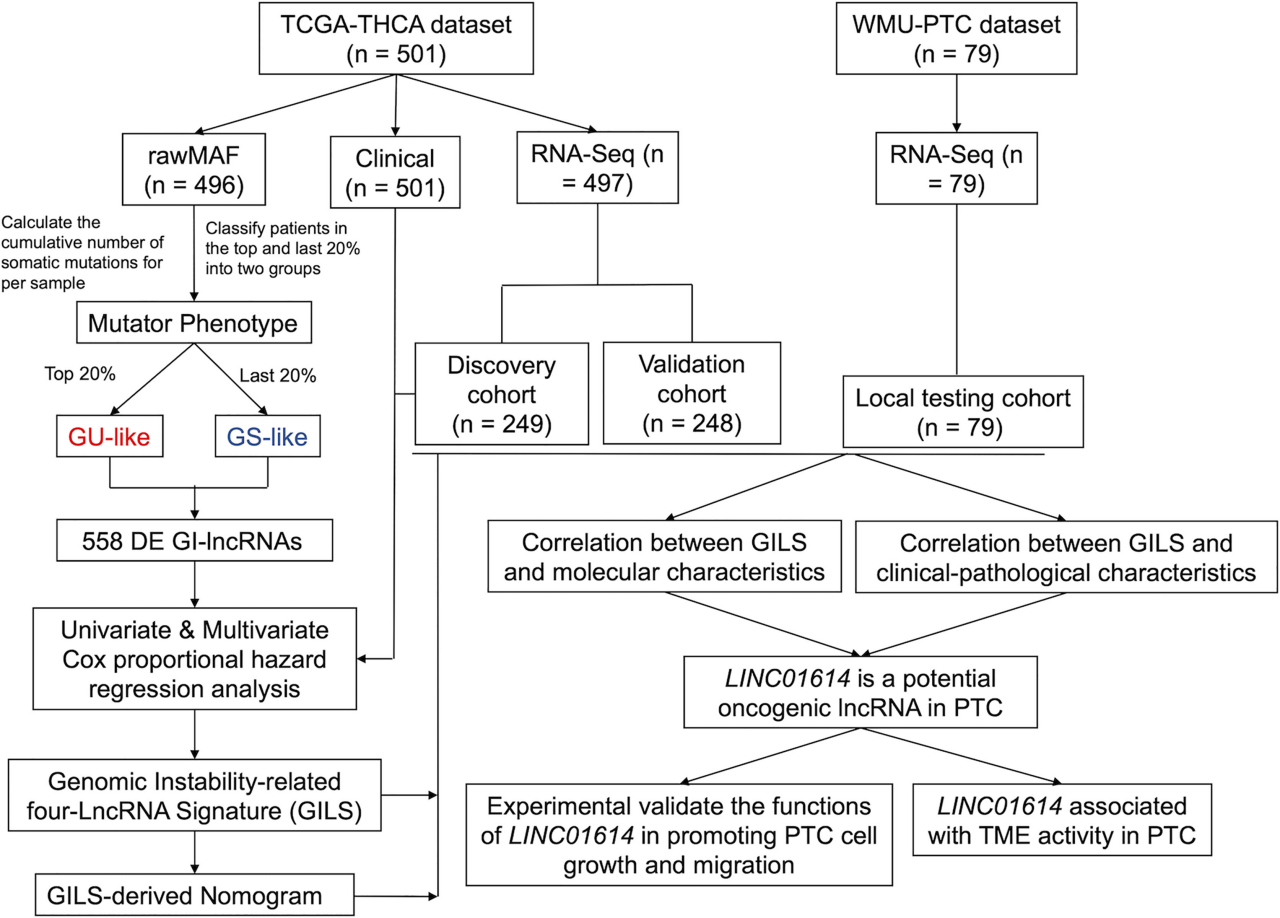
Flowchart of the present study.

**Figure 2:**
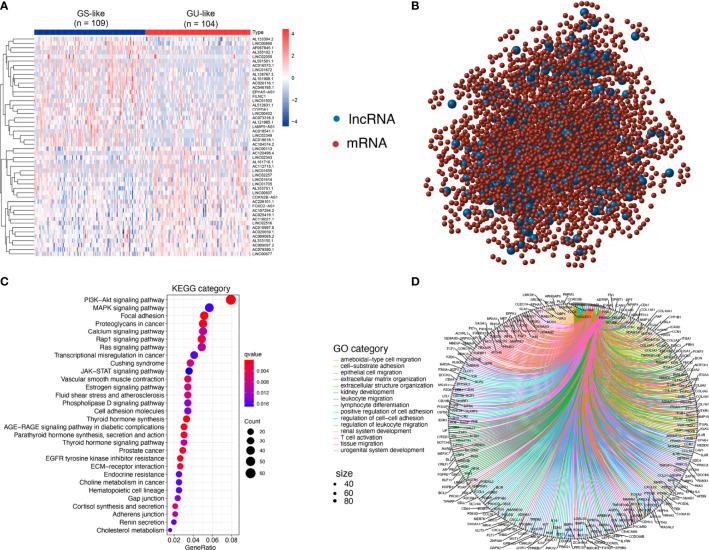
Identification and functional annotations of GIlncs in patients with PTC. **(A)** Expression patterns of 558 candidates GIlncs in 487 PTC patients. The left blue cluster is the GS-like group, and the right red cluster is the GU-like group. **(B)** Co-expression framework of GIlncs and paired mRNAs based on the Pearson correlation coefficient. The red circles represent lncRNAs, and the blue circles represent mRNAs. Functional enrichment analysis of **(C)** KEGG and **(D)** GO for mRNAs co-expressed lncRNAs.

A flowchart is shown in **Figure 1**. The cumulative number of somatic mutations in each patient was calculated and ranked in descending order. In a mutation cumulation-based method, the top 20% (n = 104) and the last 20% (n = 109) patients were assigned into the GU-like group and GS-like group, respectively. We identified 558 GIlncs were differentially expressed between the two groups (**Supplementary Table S1**), among which 533 upregulated and 25 downregulated lncRNAs in the GU-like group (**Figure 2A**).

To investigate the potential functions of the GIlncs, we explored the protein-coding genes (PCGs), which were co-expressed with lncRNA. Based on the consistency of expression, the GI-related PCGs profile was finally constitutive of the 2598 PCGs related to the lncRNA profile (**Supplementary Table S2**). The lncRNA-mRNA co-expression network was displayed (**Figure 2B**). To figure out the coupling effect between lncRNA and GI, we concentrated on the underlying functions and related pathways of these lncRNAs. Functional enrichment analysis of the GI-related PCGs was performed. KEGG pathway (**Figure 2C** and **Supplementary Table S3**) analysis revealed that significant pathways played a role in transcriptional misregulation in cancer, deregulation of cellular energy within glycolipid and choline metabolism, and endocrine resistance. Besides, enrichment of canonical pathways, such as PI3K-AKT, MAPK, and JAK-STAT pathway, provided sustained proliferative signaling. EGFR tyrosine kinase inhibitor resistance caused apoptosis inhibition GO analysis listed (**Figure 2D** and **Supplementary Table S4**) covered three aspects: biological process (BP), cellular component (CC), and molecular function (MF) in detail (25). In BP terms, the tumor microenvironment components, including leukocyte migration, T cell activation, and lymphocyte differentiation, indicated their involvement in immuno-inflammatory responses. In CC terms, migration and invasion abilities of the tumor cells were also implicated, which was shown to correlate with the extracellular matrix and cell-adhesion membrane. MF analysis stressed the significance of metabolic components such as nucleotides and polysaccharides. The above results elaborated that GIlncs lighted a novel perspective on the mechanism of PTC progression.

### Development of the GILS for Outcome Prediction

**Figure 3:**
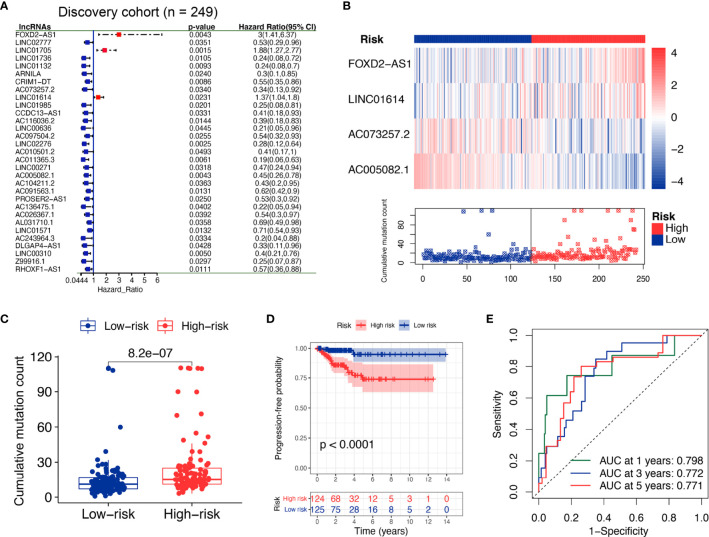
Identification of the GI-derived lncRNA signature (GILS) for outcome prediction in the discovery set. **(A)** Univariate Cox analysis of 558 candidate lncRNAs and the resultant 30 lncRNAs, shown in the forest plot with log-rank test *p*-values, hazard ratios (HR), and confidence intervals **(B)** lncRNA expression patterns and the distribution of somatic mutations. **(C)** Boxplots of somatic mutations in the GU- and GS-like group. Cumulative mutation counts in the GU-like group are significantly higher than those in the GS-like group. **(D)** Kaplan–Meier curves of PFS with low or high risk predicted by GILS in the discovery set. **(E)** Predictive accuracy of GILS for PFS. Statistical analysis was performed by log-rank test and univariate Cox proportional hazards regression analysis. PFS, progression-free survival.

The whole TCGA cohort was randomly divided into two datasets for discovery and validation. The clinical factors were not significantly different in both groups (all *p* > 0.05, **Supplementary Table S6**). The discovery set (n = 249) was used to screen clinically significant lncRNA and construct a corresponding lncRNA signature. The validation set (n = 248) was used to determine the accuracy of the signature. Univariate Cox proportional hazards regression analysis revealed that 30 lncRNAs were derived from differential lncRNAs (*p* < 0.05; **Figure 3A**). Specifically, three lncRNAs were indicated unfavorable outcomes with the HR > 1, whereas 27 lncRNAs with the HR < 1. Next, multivariate Cox proportional hazards regression analysis revealed four lncRNAs (*FOXD2-AS1*, *LINC01614*, *AC073257.2*, and *AC005082.1*) as independent prognosis biomarkers (**Supplementary Table S7**).

Based on the coefficients of multivariate analysis and lncRNAs expression, GILS was defined as follows: GILS score = 0.304 * *FOXD2-AS1* + 0.036 * *LINC01614* + (-0.769) * *AC073257.2* + (-0.328) * *AC005082.1*. Of the GILS, the coefficient of lncRNA *FOXD2-AS1* and *LINC01614* was positive, suggesting that they are risk factors, while the other lncRNA *AC073257.2* and *AC005082.1* tended to be protective factors. A comparison of individual lncRNA signature with the degree of GI demonstrated that high somatic mutation was accompanied by upregulation of *FOXD2-AS1* and *LINC01614* and downregulation of *AC073257.2* and *AC005082.1* (**Figure 3B**).

Patients in the discovery set were labeled as high- or low-risk based on the calculated GILS score, which utilized the median score as the threshold. The overall mutation rate was significantly higher in the high-risk group compared with the low-risk group (*p* = 8.2e−07; **Figure 3C**). Besides, Kaplan–Meier analysis of the discovery set showed that there was a significant difference in PFS between high and low-risk groups (*p* < 0.0001, log-rank test; **Figure 3D**). As shown in **Figure 3E**, the GILS showed an accurate estimation performance with an area under the curve (AUC) of 0.798, 0.772, and 0.771 at 1, 3, and 5 years, respectively.

### Independent Validation of GILS in the PTC Dataset

**Figure 4:**
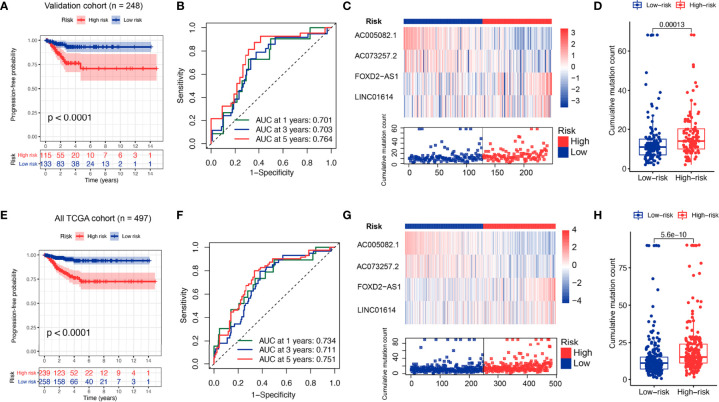
Performance evaluation of the GILS in the whole TCGA set and validation set. Kaplan–Meier curves, ROC analysis of PFS, expression of individual lncRNA as well as somatic mutations in GU- and GS-like group predicted by GILS in the validation set **(A–D)** and all TCGA set **(E–H)**. Statistical analysis was performed using the log-rank test and Cox hazards regression analysis. PFS, progression-free survival.

To verify the performance of the GILS for prognosis prediction, we calculated the GILS scores of the validation set and the entire TCGA set. Patients in the low-risk group showed a more prolonged survival than patients in the high-risk group with an AUC value of 0.764 in the validation cohort (*p* < 0.0001; **Figures 4A, B**). Similar results were also observed in the entire TCGA set, where the AUC of the ROC curves for GILS was 0.751 (*p* < 0.0001; **Figures 4E, F**). These results indicate that GILS has a good survival prediction efficacy. In both validation cohorts, *FOXD2-AS1* and *LINC01614* were upregulated, while *AC073257.2* and *AC005082.1* were down-regulated in the high-risk group (**Figures 4C, G**). Comparison analysis showed that there were significant differences in the number of somatic mutations between the high-risk and low-risk groups in both the validation (*p* < 0.0001; **Figure 4D**) and the entire TCGA cohort (*p* < 0.0001; **Figure 4H**).

### Construction and Performance Evaluation of the Nomogram

**Figure 5:**
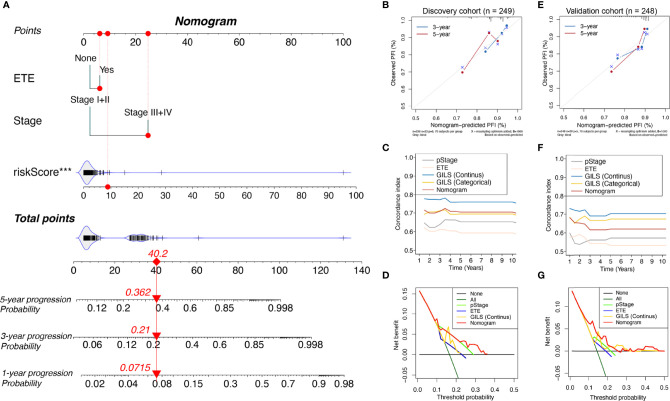
Competing-risk nomogram and the corresponding calibration. **(A)** Competing-risk nomogram incorporating the extra-thyroidal evasion, tumor stage, and GILS score. Calibration curves, concordance index estimates, and net benefit analysis of the competing-risk nomogram in the **(B-D)** discovery cohort and **(E–G)** validation cohort. ***p < 0.001.

In the discovery cohort, univariate analysis revealed the pathologic stage, ETE, and GILS score were significantly associated with PFS. To improve the clinical practicability of GILS, we established a statistical GILS-nomogram model in the discovery set by integrating GILS score, ETE, and pathologic stage (**Figure 5A**). The calibration curve showed good agreement among the estimations with the GILS-nomogram and actual observations in both discovery and validation cohorts (**Figures 5B, E**). The GILS-nomogram also yielded supreme concordance index (C-index) when compared to pathologic stage and ETE (**Figures 5C, F**). Decision curve analysis (DCA) revealed that the net benefit of the GILS-nomogram was improved when compared with other prognostic factors alone (**Figures 5D, G**). Therefore, these findings indicate improved prediction performance of the GILS-nomogram.

### Further Testing of the GILS in the WMU-PTC Cohort

**Figure 6:**
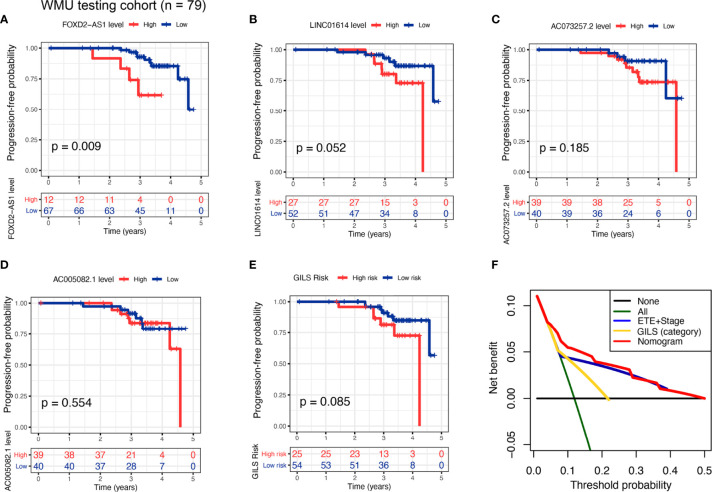
External test of the GILS using WMU-PTC cohort. Kaplan–Meier curves comparing patients with high- and low-level of **(A)** *FOXD2−AS1*, **(B)** *LINC01614*, **(C)** *AC073257.2*, and **(D)** *AC005082.1*, as well as **(E)** high- and low- GILS score along with the log-rank test. The *p*-values and HR with confidence intervals are shown for PFS in the WMU-PTC testing cohort. **(F)** Analysis of the decision curve GILS was superior to other models. PFS, progression-free survival.

To an outward promotion of our constructed GILS and GILS-nomogram, we carried out the same procedures for external testing in our cohort (n = 79). Only *FOXD-AS1* and *LINC01614* achieved satisfactory discrimination in both the high- and low-risk cohorts (*p* = 0.009 and 0.052, respectively; **Figures 6A, B**), while *AC073257.2* and *AC005082.1* had no significant difference (*p* = 0.185 and 0.554, respectively; **Figures 6C, D**). We calculate the GILS score for each patient in the WMU-PTC cohort (**Supplementary Table S5**, **9**), and we found high GILS score was associated with shorter PFS (*p* = 0.085, **Figure 6E**). As expected, the net benefit of the GILS-nomogram was highest when compared with other prognostic factors (**Figure 6F**).

### Independent Prognostic Significance of GILS and Other Clinical Factors

To verify the independent predictive effects of GILS, we combined GILS with clinical factors for univariate and multivariate Cox analysis. The results revealed that ETE, disease stage, GILS and was independent prognostic factors (**SupplementaryTable S8**). Besides, to assess the risk clustering ability of GILS in different risk stratifications, we analyzed prognostic significance of GILS in whole TCGA cohort after adjusted by clinical characteristics. There were significant difference in PFS between the young-patient (*p* < 0.0001; **Supplementary Figure S1A**) and old-patient groups (*p* < 0.0001; **Supplementary Figure S1B**); female-patient (*p* < 0.0001; **Supplementary Figure S1C**) and male-patient groups (*p* = 0.018; **Supplementary Figure S1D**); T1+T2 (*p* < 0.0001; **Figure S1E**) and T3+T4 groups (*p* = 0.007; **Supplementary Figure S1F**); Without-LNM (*p* = 0.003; **Supplementary Figure S1G**) and with-LNM groups (*p* < 0.0001; **Supplementary Figure S1H**); Early-stage (*p* < 0.0001; **Supplementary Figure S1I**) and advanced stage groups (*p* = 0.009; **Figure S1J**); Without-RT (*p* = 0.043; **Supplementary Figure S1K**) and with-RT groups (*p* < 0.0001; **Supplementary Figure S1L**). In summary, these results suggested that the prognostic significance of GILS and might widely applied in different risk stratifi;cations.

### Association Between GILS and Molecular Features of PTC

**Figure 7:**
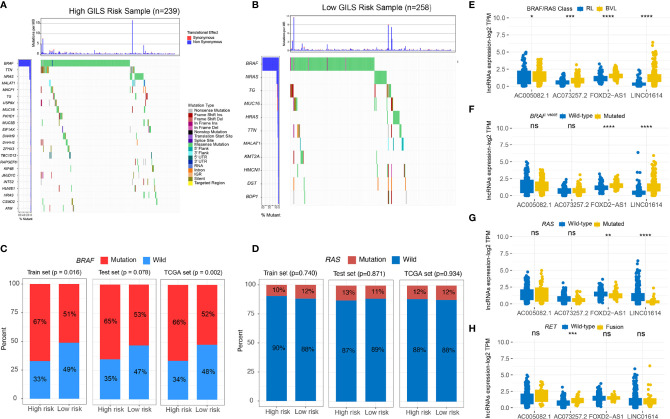
The GILS is related to the molecular characteristics of PTC. **(A, B)** The general genomic landscape is shown by waterfall plots for the GU- and GS-like groups. The proportion of mutation status of **(C)** *BRAF* and **(D)** *RAS* in different cohorts. **(E–H)** GIlncs level in different **(E)** BRAF/RAS-like phenotype and mutation status of **(F)** *BRAF* ^V600E^, **(G)** *RAS*, and **(H)** *RET* subgroups. ns, no significance, *p < 0.05, **p < 0.01, ***p < 0.001, ****p < 0.0001.

We divided the whole TCGA-PTC cohort into high and low-risk groups based on stratification by GILS score. The distinctive mutation distribution profiles of the top mutated genes in the two groups are shown (**Figures 7A, B** **)**. The MAPK and PI3K-AKT pathways play an important role in PTC malignancy. The MAPK and PI3K-AKT pathway-related mutations, *BRAF* and *RAS*, were frequently observed in both high and low-risk subgroups. We compared the mutated percent between the high-risk and low-risk groups in train, validation, and whole TCGA datasets using the chi-square test. The results showed that patients in the high-risk group displayed a significantly higher proportion of *BRAF* mutations than those in the low-risk group among the three datasets (Whole set: *p* = 0.002; discovery set: *p* = 0.016; validation set: *p* = 0.078; **Figure 7C**). However, we didn’t find a significant difference of *RAS* mutations between the two groups (**Figure 7D**). We further explored whether four GIlncs are associated with PTC molecular characteristics. The results revealed that four GIlncs were all upregulated in BVL compared to the RL group (*p* < 0.05; **Figure 7E**), especially for *FOXD2-AS1* and *LINC01614*. We also observed that the *FOXD2-AS1* and *LINC01614* were significantly upregulated in *BRAF* ^V600E^ mutation groups (*p* < 0.001; **Figure 7F**), while *FOXD2-AS1* and *LINC01614* were significantly downregulated in *RAS* mutation groups (*p* < 0.01; **Figure 7G**). Interestingly, we found *AC073257.2* was upregulated in *RET* fusion group (*p* < 0.001; **Figure 7H**). As described above, we found both GIlncs and GILS scores were correlated with critical molecular characteristics of PTC.

### GILS Associated With Clinical Parameters of PTC Patients

**Figure 8:**
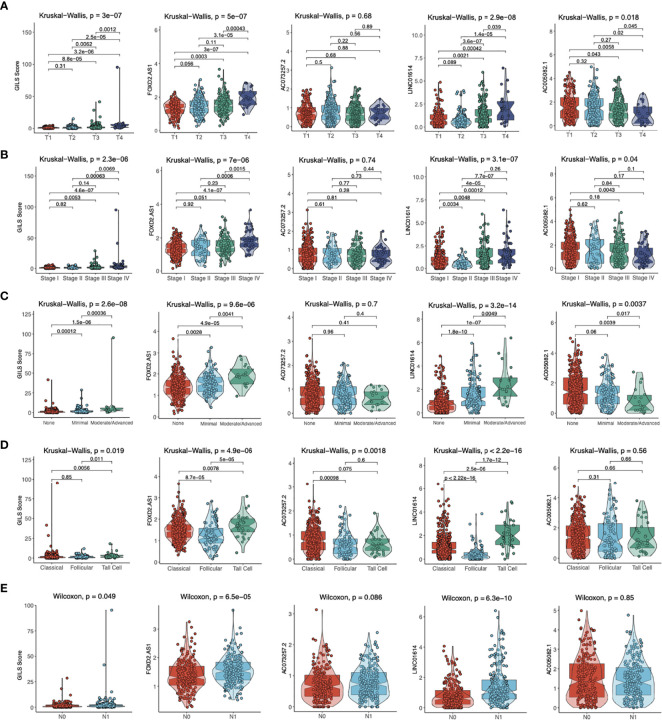
The distribution of GILS scores in relation to clinical-pathological characteristics. The difference in GILS scores among **(A)** tumor stage, **(B)** pathological stage, **(C)** extrathyroidal extension, **(D)** histological types, and **(E)** lymph node metastasis subgroups as determined by the Kruskal-Wallis test or Wilcoxon test. The thick line represents the median value, and the scattered dots represent all score values. The bottom and top levels of the boxes display 25th and 75th percentiles, respectively.

To investigate four GIlncs and GILS in PTC progression, we evaluated four GIlncs levels and GILS scores in different clinical risk stratifications. We found *FOXD2-AS1 and LINC01614* levels and GILS score were positively associated with T stage, N stage, disease stage, the degree of ETE, and histological subtypes. In addition, *AC073257.2* was differentially expressed in different histological subtypes, while *AC005082.1* was negatively associated with T stage, disease stage, and ETE (**Figures 8A-E**). These results indicated both GIlncs and GILS scores were significantly associated with the clinical outcome of PTC patients.

### Effect of *LINC01614* on PTC Cell Lines *In Vitro*

**Figure 9:**
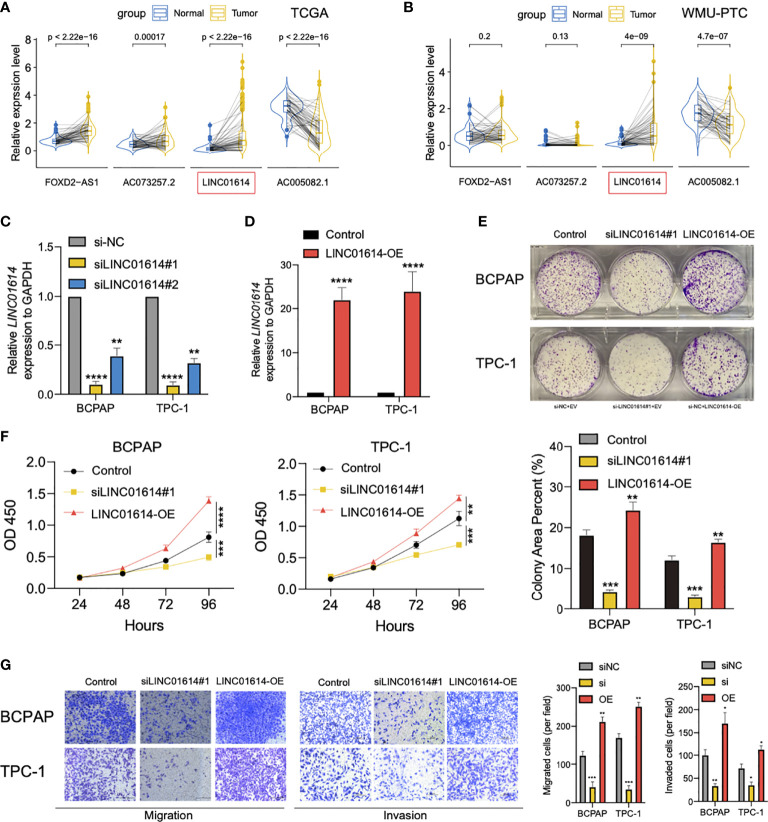
*LINC01614* behaved as an oncogene in PTC cells. The pairwise expression of individual lncRNAs in tumor and normal tissue for **(A)** TCGA cohort and **(B)** WMU-PTC cohort. **(C)** The silencing efficiency of siRNA targeting *LINC01614* was analyzed by qPCR after transfected for 48 hours in either TPC-1 or BCPAP cells. **(D)** qRT-PCR showing the relative expression of *LINC01614* in mRNA level in PTC cells transduced with empty vector or *LINC01614*-overexpressing plasmid. **(E, F)** Colony formation and CCK-8 assays were performed to test the survival of PTC cells after silencing or overexpressing of *LINC01614*. **(G)** Transwell assays were carried out to test the migration and invasive activity of *LINC01614* altered PTC cells. The results above were summarized as bar graph. All data are the means ± SD of three experiments. ^*^ *p* < 0.05, ^**^ *p* < 0.01, ^***^ *p* < 0.001, ^****^ *p* < 0.0001.

We compared the expression profiles of four GIlncs in both tumor and normal tissue in TCGA, GTEx, and WMU-PTC cohorts. In the pan-cancer analysis, we found that *FOXD2-AS1 and LINC01614* were highly expressed in most cancer types (**Supplementary Figure S2**). Besides, *FOXD2-AS1, LINC01614*, and *AC073257.2* were upregulated in PTC compared to normal tissue, while *AC005082.1* was downregulated in the TCGA cohort (**Figure 9A**). In the local cohort, we validated *LINC01614 was* upregulated in PTC compared to normal tissue, whereas *AC005082.1* was downregulated (**Figure 9B**). To explore the potential effect of *LINC01614* in PTC tumorigenesis, we silenced *LINC01614* with specific siRNA (**Figure 9C**) and overexpressed *LINC01614* with LINC01614*-*plasmid (**Figure 9D**)*. LINC01614* knockdown and overexpression significantly decreased and increased colony formation, respectively (**Figure 9E**). CCK-8 assay showed that *LINC01614* knockdown and overexpression decreased and increased PTC cell proliferation, respectively (**Figure 9F**). Furthermore, results of transwell assay (**Figure 9G**) showed that *LINC01614* depletion or overexpression in PTC cells decreased or increased cellular migration and invasion, respectively. Our results indicated that altered *LINC01614* may affect cell growth and migration in the PTC cells.

### Effect of *LINC01614* on PTC Microenvironment

**Figure 10:**
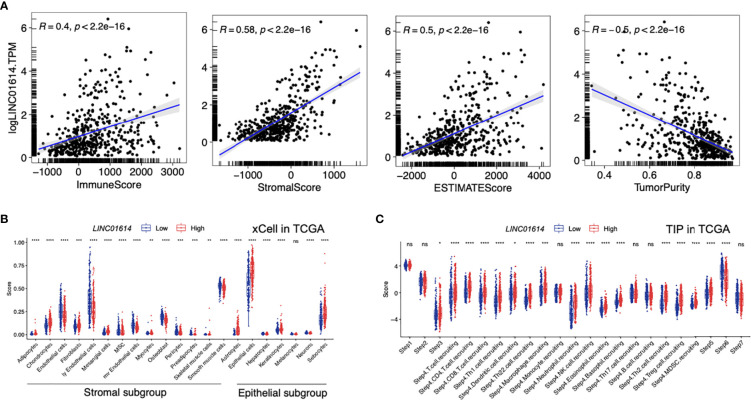
TME components in distinct *LINC01614* levels in PTC patients. **(A)** Comparison of the immune score, stromal score, microenvironment score, and tumor purity in different *LINC01614* level groups. **(B)** Comparison of the stromal and epithelial components quantified by xCell in the different *LINC01614* level groups. **(C)** Comparison of the activity of the cancer immunity cycle between the *LINC01614* ^high^ and *LINC01614* ^low^ groups. ns, no-significance, ^*^p < 0.05, ^**^p < 0.01, ^***^p < 0.001, ^****^p < 0.0001.

Furthermore, we aimed to explore the underlying correlation between the *LINC01614* mRNA level and the PTC microenvironment. As shown in **Figure 10A**, *LINC01614* was found to be positively correlated with the immune score (r = 0.4, *p* < 0.001), stromal score (r = 0.58, *p* < 0.001), and TME score (r = 0.5, *p* < 0.001), whereas negatively correlated with tumor purity (r = -0.5, *p* < 0.001). By comparing different types of non-immune TME signatures between *LINC01614* ^high^ and *LINC01614* ^low^ subgroups (**Figure 10B**), we found that the *LINC01614* ^high^ subgroup was marked with higher infiltration level of adipocytes, chondrocytes, mesangial cells, astrocytes, epithelial cells, keratinocytes, and sebocytes (All *p*-value < 0.001), whereas *LINC01614* ^low^ subgroup was marked with higher infiltration level of endothelial cells, MSC, osteoblast, smooth muscle cells, hepatocytes, and neurons (All *p*-value < 0.001).

The activities of the cancer immunity cycle are a direct comprehensive performance of the functions of the chemokine system and other immunomodulators. In the *LINC01614* ^high^ group, activities of the majority of the steps in the cycle were found to be upregulated, including priming and activation (Step 3), and trafficking of immune cells to tumors (Step 4) (CD8 T cell recruiting, Macrophage recruiting, Th1 cell recruiting, NK cell recruiting, DC recruiting, and TH17 recruiting) and infiltration of immune cells into tumors (Step 5),. Interestingly, the activity of recognition of cancer cells by T cells (Step 6) was downregulated in the *LINC01614* ^high^ group (All *p*-value < 0.001, **Figure 10C**).

## Discussion

Several clinicopathological features have been reported to be significantly associated with poor prognosis in PTC patients, including larger primary tumor diameter, older age at first diagnosis, extrathyroidal invasion, lymph node metastasis, and distant metastasis (26). TNM staging and other scoring systems have been used to predict the clinical outcome of PTC patients (27, 28). However, the current acknowledged mortality risk-stratification system for PTC, which integrated numerous clinicopathological parameters, seemed uninformative for the foresight of recurrence risk (29–31). Besides, patients with similar clinical phenotypes do not have identical prognoses, suggesting that the present PTC prognostic evaluation system may not provide an accurate clinical prognosis for every patient. Therefore, the PTC prognostic evaluation system needs improvement, and the accuracy of PTC prognoses could be significantly improved using molecular biomarkers. Over the past decade, accumulating researches have demonstrated that lncRNAs played essential roles in multiple biological events, such as maintaining genomic stability (32). Cancer patients with a higher GI level have a worse clinical prognosis, so GI could be used to evaluate the clinical prognosis of tumor patients (33). In recent years, lncRNAs have also been shown to be involved in genome stability, and no relevant studies have investigated the lncRNA signatures of GI in PTC. For example, aberrant expression of lncRNAs could affect the development and progression of malignant tumors (34). Therefore, we constructed a novel signature of GIlncs in PTC and explored their significance in predicting the patient’s prognosis.

Numerous genomic classifiers have been used to predict the clinical outcome of PTC. For example, the ThyroSeq v3 classifier integrated the mutational information of 112 genes, a collection of gene fusions, and the expression profile of 19 DEGs (35). As for non-coding genes, miRNAs were proposed as the regulators of the essential phenotypes of PTC by fine-tuning gene expression (signaling, differentiation, and invasion process) (1, 36). By comparison, few classifiers shed light on the significance of lncRNAs in the prognosis of PTC. Unlike miRNAs that acted on major post-transcriptional mechanisms, lncRNAs were able to combine with homologous nucleotide sequences (37). According to our results of functional enrichment analysis, it could be inferred that lncRNA signatures were involved in the cancer hallmarks as described above, which provided sufficient support for the predictive role of GI. Additionally, the complexity of spatial folding configurations endowed lncRNAs with the binding sites, which could combine certain macromolecular proteins. lncRNAs with aberrant expression may dampen the cancer genome’s stability by exerting coupling effects to disrupt the regulation of lncRNA-related PCGs. Significantly differentially expressed lncRNAs are thus, profiled as predictors of genome instability.

Recently, an increasing number of studies have revealed the potential links between lncRNA and GI. It was noteworthy that over the past decades, increasing studies of lncRNA in the prognosis of human cancers were published through the organic combination with GI (14, 38–41). For instance, analogous analysis in breast cancer illustrated a novel panel of lncRNAs as GI-associated tumor biomarkers (16). The GILS model could stratify risk subgroups based on the degree of GI and serve as a robust prognostic index independently. With the development of the function of lncRNA binding sites and their role in genomic variation, increasing researchers have paid attention to the mechanisms of lncRNA in cancer. Up to now, it has been proved that certain lncRNAs were involved in tumorigenesis by promoting DNA damage, altering drug metabolism, regulating cell apoptosis, affecting the EMT process (6, 14, 42–44).

Currently, there is no effective biomarker to predict the prognosis of PTC. We constructed a GILS model as an independent prognostic predictor based on multivariate Cox regression analysis and exhibited the prediction model using a visual nomogram. In our nomogram, ETE and tumor stage were utilized as binary variables, while the GILS score was utilized as a continuous variable. Other variables significantly related to the clinical prognosis of PTC will also be included in the future prediction model. To assess the clinical significance of this competing-risk nomogram, we performed the net benefits analysis using DCA. As a new analysis for evaluating the clinical prediction model, DCA could integrate the preferences of patients or decision-makers into the analysis (45). By calculating the net benefits at different threshold probabilities, we found that the GILS-nomogram had a satisfactory net benefit, which potentially implied clinical practice.

The current study investigated the role of lncRNAs in GI and identified four lncRNAs as independent prognostic biomarkers for PTC. First, we selected 558 lncRNAs differentially expressed in GS-like and GU-like phenotypes, which were enriched in GI-related pathways based on the functional enrichment analysis with the lncRNA-derived mRNAs. Next, the prognostic significance of GI-derived lncRNA biomarkers was identified after the univariate and multivariate Cox regression analyses. Further internal validation emphasized the general applicability of these biomarkers. Many studies have demonstrated that some GILS-derived lncRNAs were associated with specific cancers. In other cases, *FOXD2-AS1* had been identified as an oncogene promoting the progression of different cancers, such as PTC and bladder cancer (46–48). *FOXD2-AS1* also enhanced chemotherapeutic resistance in esophageal and laryngeal squamous cell carcinoma (49, 50). *FOXD2-AS1* was recognized as a molecular biomarker and participated in chemo-resistance, malignant proliferation, invasion, and immunosuppression (46, 51–55). Recently, a study demonstrated that overexpression of *FOXD2-AS1* was associated with poor clinical outcomes in PTC patients, and the knockdown of *FOXD2-AS1* could suppress tumor growth by reversing its sponge effect (56). However, *AC073257.2* and *AC005082.1* have not been reported in cancer yet. The mechanisms of their function in cancer require further research. Furthermore, we observed that GILS could identify the *BRAF* mutation status and share commonalities with clinical prognostic parameters. In addition, lncRNA, mRNA & miRNA likely work together to form a competing endogenous RNA (ceRNA) regulatory network, which could be used as prognostic markers in cancers (57–59). GI-associated ceRNA regulatory network should be evaluated in the future studies.

A pan-cancer analysis only briefly demonstrated that the *LINC01614* was upregulated in different cancers, but no further in-depth analysis was performed in PTC (60). Another research found that *LINC01614* could serve as an emulative sponge to combine with miR-383 and result in aggressive behavior in glioma (61). In esophageal squamous cell carcinoma and breast cancer, *LINC01614* was also suggested as a survival predictor and had an impact on tumor invasion (62, 63). However, the functions of *LINC01614* are not clear in PTC. We found that *LINC01614* is significantly overexpressed in PTC compared with the non-tumor thyroid tissues in both TCGA and local cohorts. In experiments, silencing of *LINC01614* significantly inhibited survival of PTC cells while over-expressing of *LINC01614* reverse it. In addition, Transwell analysis indicated that *LINC01614* promotes PTC cells migration and invasion abilities. Both immune cell and stromal cell exerts huge to regulate tumor progression, and TME activity is closely associated with genomic instability (64, 65). ESTIMATE and xCell analysis suggested *LINC01614* closely associated with infiltration level of stromal and epithelial cells. TIP analysis demonstrated *LINC01614* closely associated with cancer-immunity cycle in PTC.

This study has several limitations. First, the number of patients in our local testing cohort is still small, and the median follow-up period wasn’t long enough. Thus, additional larger cohorts are required to validate these findings. Second, although we investigated the potential value of GILS using bioinformatics analysis and using local testing cohort, further testing is still lacking in external datasets. Third, the potential ceRNA-regulated mechanism of *LINC01614* in PTC progression needs further investigation. Lastly, we only verified that *LINC01614* is an oncogene, but its oncogenic function whether related to genomic instability should be explored.

In conclusion, we screened out GIlncs and constructed a corresponding lncRNA signature. Four GIlncs were identified as independent prognosis factors. Based on this, we established a composite GILS-nomogram for PTC patients to predict the clinical outcome, which was verified in internal validation and our local cohort. This GILS model could identify high-risk PTC patients and precisely formulate treatment strategies, which could potentially benefit the management of PTC patients. These findings not only suggested that the GILS might serve as the biomarker for pathological classification system and prognosis prediction but demonstrated *LINC01614* as a novel oncogenic TME-related lncRNA in PTC.

## Data Availability Statement

The datasets presented in this article are not readily available because the local RNA-sequencing data used in this paper belongs to a larger unpublished clinical research project. According to the terms of the project contract, the complete sequencing data will be uploaded to the GEO when the main articles are published. This paper is a part of the project: Major Science and Technology Projects of Zhejiang Province, 2015C03052. Requests to access the datasets should be directed to the corresponding author(s).

## Ethics Statement

The studies involving human participants were reviewed and approved by All research protocols have been approved and implemented through the ethical standards of the institutional review board of the First Affiliated Hospital of Wenzhou Medical University (Approval No. 2012-57). The patients/participants provided their written informed consent to participate in this study.

## Author Contributions

XD contributed to study design, bioinformatic analysis, and manuscript editing. XD, YC, and Z-qY contributed to the manuscript draft. DC and CJ collected and analyzed clinical samples for the study. XH, XZ, WZ, and D-nG discussed the results and participated in the critical review of the manuscript. All authors contributed to the article and approved the submitted version.

## Funding

This study was supported by the funding of the National Natural Science Foundation of China (No. 81802328), Major Science and Technology Projects of Zhejiang Province (2015C03052), and Young Talents Program of the First Affiliated Hospital of Wenzhou Medical University (No. qnyc094).

## Conflict of Interest

The authors declare that the research was conducted in the absence of any commercial or financial relationships that could be construed as a potential conflict of interest.

## Publisher’s Note

All claims expressed in this article are solely those of the authors and do not necessarily represent those of their affiliated organizations, or those of the publisher, the editors and the reviewers. Any product that may be evaluated in this article, or claim that may be made by its manufacturer, is not guaranteed or endorsed by the publisher.

## Supplementary Material

Supplementary Figure 1 Patient stratification based on key clinical-pathological parameters. Kaplan–Meier estimates of PFS in high- and low-risk groups of patients with different **(A–B)** age, **(C–D)** gender, **(E–F)** tumor size, **(G–H)** lymph nodes involvement, **(I–J)** clinical stage, and **(K–L)** radiation therapy status. Statistical analysis was performed using the log-rank test and univariate Cox analysis. PFS, progression-free survival.

Supplementary Figure 2 The expression pattern of *FOXD2-AS1*, *LINC01614*, *AC073257.2* and *AC005082.1* of pan-cancers in TCGA combined with GTEx.

## References

[1] Cancer Genome Atlas Research N. Integrated Genomic Characterization of Papillary Thyroid Carcinoma. Cell (2014) 159(3):676–90. doi: 10.1016/j.cell.2014.09.050 PMC4243044 25417114

[2] Oliva-Rico D Herrera LA. Regulated Expression of the lncRNA TERRA and its Impact on Telomere Biology. Mech Ageing Dev (2017) 167:16–23. doi: 10.1016/j.mad.2017.09.001 28888705

[3] Siegel RL Miller KD Jemal A. Cancer Statistics, 2020. CA Cancer J Clin (2020) 70(1):7–30. doi: 10.3322/caac.21590 31912902

[4] Grogan RH Kaplan SP Cao H Weiss RE Degroot LJ Simon CA. A Study of Recurrence and Death From Papillary Thyroid Cancer With 27 Years of Median Follow-Up. Surgery (2013) 154(6):1436–46. doi: 10.1016/j.surg.2013.07.008 24075674

[5] Schneider DF Chen H. New Developments in the Diagnosis and Treatment of Thyroid Cancer. CA Cancer J Clin (2013) 63(6):374–94. doi: 10.3322/caac.21195 PMC3800231 23797834

[6] Lecerf C Le Bourhis X Adriaenssens E. The Long Non-Coding RNA H19: An Active Player With Multiple Facets to Sustain the Hallmarks of Cancer. Cell Mol Life Sci (2019) 76(23):4673–87. doi: 10.1007/s00018-019-03240-z PMC11105575 31338555

[7] Hanahan D Weinberg RA. The Hallmarks of Cancer. Cell (2000) 100(1):57–70. doi: 10.1016/s0092-8674(00)81683-9 10647931

[8] Nakashima M Suzuki K Meirmanov S Naruke Y Matsuu-Matsuyama M Shichijo K. Foci Formation of P53-Binding Protein 1 in Thyroid Tumors: Activation of Genomic Instability During Thyroid Carcinogenesis. Int J Cancer (2008) 122(5):1082–8. doi: 10.1002/ijc.23223 17985346

[9] Iyer MK Niknafs YS Malik R Singhal U Sahu A Hosono Y. The Landscape of Long Noncoding RNAs in the Human Transcriptome. Nat Genet (2015) 47(3):199–208. doi: 10.1038/ng.3192 25599403 PMC4417758

[10] Huarte M. The Emerging Role of lncRNAs in Cancer. Nat Med (2015) 21(11):1253–61. doi: 10.1038/nm.3981 26540387

[11] Lee S Kopp F Chang TC Sataluri A Chen B Sivakumar S. Noncoding RNA NORAD Regulates Genomic Stability by Sequestering PUMILIO Proteins. Cell (2016) 164(1-2):69–80. doi: 10.1016/j.cell.2015.12.017 26724866 PMC4715682

[12] Betts JA Moradi Marjaneh M Al-Ejeh F Lim YC Shi W Sivakumaran H. Long Noncoding RNAs CUPID1 and CUPID2 Mediate Breast Cancer Risk at 11q13 by Modulating the Response to DNA Damage. Am J Hum Genet (2017) 101(2):255–66. doi: 10.1016/j.ajhg.2017.07.007 PMC5544418 28777932

[13] Polo SE Blackford AN Chapman JR Baskcomb L Gravel S Rusch A. Regulation of DNA-End Resection by hnRNPU-Like Proteins Promotes DNA Double-Strand Break Signaling and Repair. Mol Cell (2012) 45(4):505–16. doi: 10.1016/j.molcel.2011.12.035 PMC3550743 22365830

[14] Hu Z Mi S Zhao T Peng C Peng Y Chen L. BGL3 lncRNA Mediates Retention of the BRCA1/BARD1 Complex at DNA Damage Sites. EMBO J (2020) 39(12):e104133. doi: 10.15252/embj.2019104133 32347575 PMC7298298

[15] Guo F Li L Yang W Hu J-f Cui J. Long Noncoding RNA: A Resident Staff of Genomic Instability Regulation in Tumorigenesis. Cancer Lett (2021) 503:103–9. doi: 10.1016/j.canlet.2021.01.021 33516792

[16] Bao S Zhao H Yuan J Fan D Zhang Z Su J. Computational Identification of Mutator-Derived lncRNA Signatures of Genome Instability for Improving the Clinical Outcome of Cancers: A Case Study in Breast Cancer. Brief Bioinform (2020) 21(5):1742–55. doi: 10.1093/bib/bbz118 31665214

[17] Geng W Lv Z Fan J Xu J Mao K Yin Z. Identification of the Prognostic Significance of Somatic Mutation-Derived lncRNA Signatures of Genomic Instability in Lung Adenocarcinoma. Front Cell Dev Biol (2021) 9:736. doi: 10.3389/fcell.2021.657667 PMC8039462 33855028

[18] Guo M Wang SM. Genome Instability-Derived Genes Are Novel Prognostic Biomarkers for Triple-Negative Breast Cancer. Front Cell Dev Biol (2021) 9:1753. doi: 10.3389/fcell.2021.701073 PMC8312551 34322487

[19] Yang H Xiong X Li H. Development and Interpretation of a Genomic Instability Derived lncRNAs Based Risk Signature as a Predictor of Prognosis for Clear Cell Renal Cell Carcinoma Patients. Front Oncol (2021) 11:1634. doi: 10.3389/fonc.2021.678253 PMC8176022 34094983

[20] Bao S Hu T Liu J Su J Sun J Ming Y. Genomic Instability-Derived Plasma Extracellular vesicle-microRNA Signature as a Minimally Invasive Predictor of Risk and Unfavorable Prognosis in Breast Cancer. J Nanobiotechnol (2021) 19(1):22. doi: 10.1186/s12951-020-00767-3 PMC7802300 33436002

[21] Colaprico A Silva TC Olsen C Garofano L Cava C Garolini D. TCGAbiolinks: An R/Bioconductor Package for Integrative Analysis of TCGA Data. Nucleic Acids Res (2016) 44(8):e71–e. doi: 10.1093/nar/gkv1507 PMC4856967 26704973

[22] Liu J Lichtenberg T Hoadley KA Poisson LM Lazar AJ Cherniack AD. An Integrated TCGA Pan-Cancer Clinical Data Resource to Drive High-Quality Survival Outcome Analytics. Cell (2018) 173(2):400–16.e11. doi: 10.1016/j.cell.2018.02.052 29625055 PMC6066282

[23] Dong X Yang Q Gu J Lv S Song D Chen D. Identification and Validation of L Antigen Family Member 3 as an Immune-Related Biomarker Associated With the Progression of Papillary Thyroid Cancer. Int Immunopharmacol (2020) 90:107267. doi: 10.1016/j.intimp.2020.107267 33310661

[24] Aran D Hu Z Butte AJ. Xcell: Digitally Portraying the Tissue Cellular Heterogeneity Landscape. Genome Biol (2017) 18(1):1–14. doi: 10.1186/s13059-017-1349-1 29141660 PMC5688663

[25] Maurano MT Humbert R Rynes E Thurman RE Haugen E Wang H. Systematic Localization of Common Disease-Associated Variation in Regulatory DNA. Science (2012) 337(6099):1190–5. doi: 10.1126/science.1222794 PMC3771521 22955828

[26] Randolph GW Duh QY Heller KS LiVolsi VA Mandel SJ Steward DL. The Prognostic Significance of Nodal Metastases From Papillary Thyroid Carcinoma can be Stratified Based on the Size and Number of Metastatic Lymph Nodes, as Well as the Presence of Extranodal Extension. Thyroid (2012) 22(11):1144–52. doi: 10.1089/thy.2012.0043 23083442

[27] Cabanillas ME McFadden DG Durante C. Thyroid Cancer. Lancet (2016) 388(10061):2783–95. doi: 10.1016/S0140-6736(16)30172-6 27240885

[28] Gong W Yang S Yang X Guo F. Blood Preoperative Neutrophil-to-Lymphocyte Ratio is Correlated With TNM Stage in Patients With Papillary Thyroid Cancer. Clinics (2016) 71(6):311–4. doi: 10.6061/clinics/2016(06)04 PMC4930667 27438563

[29] Tuttle RM Alzahrani AS. Risk Stratification in Differentiated Thyroid Cancer: From Detection to Final Follow-Up. J Clin Endocrinol Metab (2019) 104(9):4087–100. doi: 10.1210/jc.2019-00177 PMC6684308 30874735

[30] Sorrenti S Carbotta G Di Matteo FM Catania A Pironi D Tartaglia F. Evaluation of Clinicopathological and Molecular Parameters on Disease Recurrence of Papillary Thyroid Cancer Patient: A Retrospective Observational Study. Cancers (Basel) (2020) 12(12):3637. doi: 10.3390/cancers12123637 PMC7761952 33291668

[31] Lamartina L Grani G Arvat E Nervo A Zatelli MC Rossi R. 8th Edition of the AJCC/TNM Staging System of Thyroid Cancer: What to Expect (ITCO#2). Endocr Relat Cancer (2018) 25(3):L7–L11. doi: 10.1530/ERC-17-0453 29192093

[32] Böhmdorfer G Wierzbicki AT. Control of Chromatin Structure by Long Noncoding RNA. Trends Cell Biol (2015) 25(10):623–32. doi: 10.1016/j.tcb.2015.07.002 PMC4584417 26410408

[33] Genga KR Rocha Filho FD de Almeida Ferreira FV de Sousa JC Studart FS Magalhães SMM. Proteins of the Mitotic Checkpoint and Spindle are Related to Chromosomal Instability and Unfavourable Prognosis in Patients With Myelodysplastic Syndrome. J Clin Pathol (2015) 68(5):381–7. doi: 10.1136/jclinpath-2014-202728 25637637

[34] Sanchez Calle A Kawamura Y Yamamoto Y Takeshita F Ochiya T. Emerging Roles of Long non-Coding RNA in Cancer. Cancer Sci (2018) 109(7):2093–100. doi: 10.1111/cas.13642 PMC6029823 29774630

[35] Nikiforova MN Mercurio S Wald AI Barbi de Moura M Callenberg K Santana-Santos L. Analytical Performance of the ThyroSeq V3 Genomic Classifier for Cancer Diagnosis in Thyroid Nodules. Cancer (2018) 124(8):1682–90. doi: 10.1002/cncr.31245 PMC5891361 29345728

[36] Ramírez-Moya J Santisteban P. miRNA-Directed Regulation of the Main Signaling Pathways in Thyroid Cancer. Front Endocrinol (2019) 10:430. doi: 10.3389/fendo.2019.00430 PMC6614345 31312183

[37] Hon CC Ramilowski JA Harshbarger J Bertin N Rackham OJ Gough J. An Atlas of Human Long non-Coding RNAs With Accurate 5’ Ends. Nature (2017) 543(7644):199–204. doi: 10.1038/nature21374 28241135 PMC6857182

[38] Sharma V Misteli T. Non-Coding RNAs in DNA Damage and Repair. FEBS Lett (2013) 587(13):1832–9. doi: 10.1016/j.febslet.2013.05.006 PMC3710463 23684639

[39] Zhang C Peng G. Non-Coding RNAs: An Emerging Player in DNA Damage Response. Mutat Res Rev Mutat Res (2015) 763:202–11. doi: 10.1016/j.mrrev.2014.11.003 25795121

[40] Tehrani SS Karimian A Parsian H Majidinia M Yousefi B. Multiple Functions of Long Non-Coding RNAs in Oxidative Stress, DNA Damage Response and Cancer Progression. J Cell Biochem (2018) 119(1):223–36. doi: 10.1002/jcb.26217 28608608

[41] Athie A Marchese FP Gonzalez J Lozano T Raimondi I Juvvuna PK. Analysis of Copy Number Alterations Reveals the lncRNA ALAL-1 as a Regulator of Lung Cancer Immune Evasion. J Cell Biol (2020) 219(9):e201908078. doi: 10.1083/jcb.201908078 32858747 PMC7480115

[42] Merry CR Forrest ME Sabers JN Beard L Gao XH Hatzoglou M. DNMT1-Associated Long Non-Coding RNAs Regulate Global Gene Expression and DNA Methylation in Colon Cancer. Hum Mol Genet (2015) 24(21):6240–53. doi: 10.1093/hmg/ddv343 PMC4599679 26307088

[43] Sharma V Khurana S Kubben N Abdelmohsen K Oberdoerffer P Gorospe M. A BRCA1-Interacting lncRNA Regulates Homologous Recombination. EMBO Rep (2015) 16(11):1520–34. doi: 10.15252/embr.201540437 PMC4641504 26412854

[44] D’Alessandro G Whelan DR Howard SM Vitelli V Renaudin X Adamowicz M. BRCA2 Controls DNA : RNA Hybrid Level at DSBs by Mediating RNase H2 Recruitment. Nat Commun (2018) 9(1):5376. doi: 10.1038/s41467-018-07799-2 30560944 PMC6299093

[45] Vickers AJ Elkin EB. Decision Curve Analysis: A Novel Method for Evaluating Prediction Models. Med Decision Making (2006) 26(6):565–74. doi: 10.1177/0272989X06295361 PMC2577036 17099194

[46] Xu TP Wang WY Ma P Shuai Y Zhao K Wang YF. Upregulation of the Long Noncoding RNA FOXD2-AS1 Promotes Carcinogenesis by Epigenetically Silencing EphB3 Through EZH2 and LSD1, and Predicts Poor Prognosis in Gastric Cancer. Oncogene (2018) 37(36):5020–36. doi: 10.1038/s41388-018-0308-y 29789713

[47] Su F He W Chen C Liu M Liu H Xue F. The Long Non-Coding RNA FOXD2-AS1 Promotes Bladder Cancer Progression and Recurrence Through a Positive Feedback Loop With Akt and E2F1. Cell Death Dis (2018) 9(2):1–17. doi: 10.1038/s41419-018-0275-9 29445134 PMC5833400

[48] Li H Han Q Chen Y Chen X Ma R Chang Q. Upregulation of the Long non-Coding RNA FOXD2-AS1 is Correlated With Tumor Progression and Metastasis in Papillary Thyroid Cancer. Am J Trans Res (2019) 11(9):5457. PMC6789238 31632522

[49] Xue W Shen Z Li L Zheng Y Yan D Kan Q. Long Non-Coding RNAs MACC1-AS1 and FOXD2-AS1 Mediate NSD2-Induced Cisplatin Resistance in Esophageal Squamous Cell Carcinoma. Mol Therapy-Nucleic Acids (2020) 23:590–692. doi: 10.1016/j.omtn.2020.12.007 PMC7819824 33552680

[50] Li R Chen S Zhan J Li X Liu W Sheng X. Long Noncoding RNA FOXD2-AS1 Enhances Chemotherapeutic Resistance of Laryngeal Squamous Cell Carcinoma *via* STAT3 Activation. Cell Death Dis (2020) 11(1):1–13. doi: 10.1038/s41419-020-2232-7 31959918 PMC6971019

[51] Li R Chen S Zhan J Li X Liu W Sheng X. Long Noncoding RNA FOXD2-AS1 Enhances Chemotherapeutic Resistance of Laryngeal Squamous Cell Carcinoma *via* STAT3 Activation. Cell Death Dis (2020) 11(1):41. doi: 10.1038/s41419-020-2232-7 31959918 PMC6971019

[52] Su F He W Chen C Liu M Liu H Xue F. The Long Non-Coding RNA FOXD2-AS1 Promotes Bladder Cancer Progression and Recurrence Through a Positive Feedback Loop With Akt and E2F1. Cell Death Dis (2018) 9(2):233. doi: 10.1038/s41419-018-0275-9 29445134 PMC5833400

[53] Zhao J Zeng XB Zhang HY Xiang JW Liu YS. Long non-Coding RNA FOXD2-AS1 Promotes Cell Proliferation, Metastasis and EMT in Glioma by Sponging miR-506-5p. Open Med (Wars) (2020) 15(1):921–31. doi: 10.1515/med-2020-0175 PMC7711959 33336050

[54] Hu W Feng H Xu X Huang X Huang X Chen W. Long Noncoding RNA FOXD2-AS1 Aggravates Hepatocellular Carcinoma Tumorigenesis by Regulating the miR-206/MAP3K1 Axis. Cancer Med (2020) 9(15):5620–31. doi: 10.1002/cam4.3204 PMC7402827 32558350

[55] Cao L Wang Y Wang Q Huang J. LncRNA FOXD2-AS1 Regulates Chondrocyte Proliferation in Osteoarthritis by Acting as a Sponge of miR-206 to Modulate CCND1 Expression. BioMed Pharmacother (2018) 106:1220–6. doi: 10.1016/j.biopha.2018.07.048 30119190

[56] Li H Han Q Chen Y Chen X Ma R Chang Q. Upregulation of the Long Non-Coding RNA FOXD2-AS1 is Correlated With Tumor Progression and Metastasis in Papillary Thyroid Cancer. Am J Transl Res (2019) 11(9):5457–71. PMC6789238 31632522

[57] Zhang D-D Shi Y Liu J-B Yang X-L Xin R Wang H-M. Construction of a Myc-Associated ceRNA Network Reveals a Prognostic Signature in Hepatocellular Carcinoma. Mol Therapy-Nucleic Acids (2021) 24:1033–50. doi: 10.1016/j.omtn.2021.04.019 PMC8167205 34141458

[58] Ping Y Zhou Y Hu J Pang L Xu C Xiao Y. Dissecting the Functional Mechanisms of Somatic Copy-Number Alterations Based on Dysregulated ceRNA Networks Across Cancers. Mol Therapy-Nucleic Acids (2020) 21:464–79. doi: 10.1016/j.omtn.2020.06.012 PMC7358224 32668393

[59] Zhou B Ge Y Shao Q Yang L Chen X Jiang G. Long Noncoding RNA LINC00284 Facilitates Cell Proliferation in Papillary Thyroid Cancer via Impairing miR-3127-5p Targeted E2F7 Suppression. Cell Death Discov (2021) 7(1):156. doi: 10.1038/s41420-021-00551-8 34226533 PMC8257569

[60] Wang D Zhang H Fang X Cao D Liu H. Pan-Cancer Analysis Reveals the Role of Long non-Coding RNA LINC01614 as a Highly Cancer-Dependent Oncogene and Biomarker. Oncol Lett (2020) 20(2):1383–99. doi: 10.3892/ol.2020.11648 PMC7377058 32724381

[61] Wang H Wu J Guo W. SP1-Mediated Upregulation of lncRNA LINC01614 Functions a ceRNA for miR-383 to Facilitate Glioma Progression Through Regulation of ADAM12. Onco Targets Ther (2020) 13:4305–18. doi: 10.2147/OTT.S242854 PMC7244248 32547064

[62] Wang Y Song B Zhu L Zhang X. Long Non-Coding RNA, LINC01614 as a Potential Biomarker for Prognostic Prediction in Breast Cancer. PeerJ (2019) 7:e7976. doi: 10.7717/peerj.7976 31741788 PMC6858983

[63] Tang L Chen Y Peng X Zhou Y Jiang H Wang G. Identification and Validation of Potential Pathogenic Genes and Prognostic Markers in ESCC by Integrated Bioinformatics Analysis. Front Genet (2020) 11:521004. doi: 10.3389/fgene.2020.521004 33362844 PMC7758294

[64] Means C Clayburgh DR Maloney L Sauer D Taylor MH Shindo ML. Tumor Immune Microenvironment Characteristics of Papillary Thyroid Carcinoma are Associated With Histopathological Aggressiveness and BRAF Mutation Status. Head Neck (2019) 41(8):2636–46. doi: 10.1002/hed.25740 30896061

[65] Erkes DA Cai W Sanchez IM Purwin TJ Rogers C Field CO. Mutant BRAF and MEK Inhibitors Regulate the Tumor Immune Microenvironment *via* pyroptosis. Cancer Discovery (2020) 10(2):254–69. doi: 10.1158/2159-8290.CD-19-0672 PMC7007378 31796433

