# Supplementary material for: Genomic Instability-Related LncRNA Signature Predicts the Prognosis and Highlights LINC01614 Is a Tumor Microenvironment-Related Oncogenic lncRNA of Papillary Thyroid Carcinoma
Source: Front Oncol. 2021 Sep 16;11:737867. doi: 10.3389/fonc.2021.737867 (PMC8481916; doi:10.3389/fonc.2021.737867)
Supplement: Supplementary Figure 1 — Patient stratification based on key clinical-pathological parameters. Kaplan–Meier estimates of PFS in high- and low-risk groups of patients with different (A–B) age, (C–D) gender, (E–F) tumor size, (G–H) lymph nodes involvement, (I–J) clinical stage, and (K–L) radiation therapy status. Statistical analysis was performed using the log-rank test and univariate Cox analysis. PFS, progression-free survival. [file Image_1.pdf]

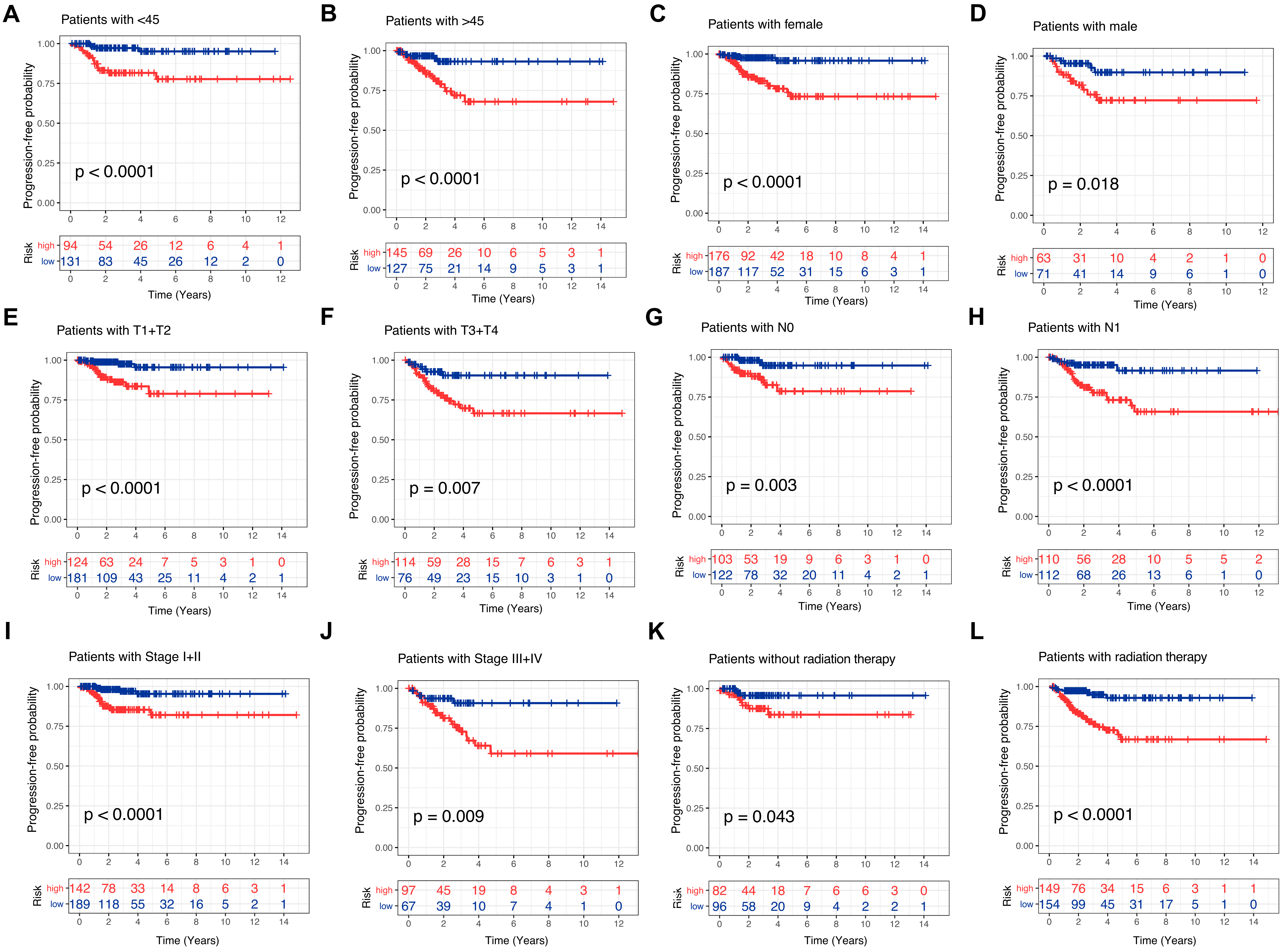

A
Patients with <45
Progression-free probability
p < 0.0001
Risk
high 94 54 26 12 6 4 1
low 131 83 45 26 12 2 0
Time (Years)
B
Patients with >45
Progression-free probability
p < 0.0001
Risk
high 145 69 26 10 6 5 3 1
low 127 75 21 14 9 5 3 1
Time (Years)
C
Patients with female
Progression-free probability
p < 0.0001
Risk
high 176 92 42 18 10 8 4 1
low 187 117 52 31 15 6 3 1
Time (Years)
D
Patients with male
Progression-free probability
p = 0.018
Risk
high 63 31 10 4 2 1 0
low 71 41 14 9 6 1 0
Time (Years)
E
Patients with T1+T2
Progression-free probability
p < 0.0001
Risk
high 124 63 24 7 5 3 1 0
low 181 109 43 25 11 4 2 1
Time (Years)
F
Patients with T3+T4
Progression-free probability
p = 0.007
Risk
high 114 59 28 15 7 6 3 1
low 76 49 23 15 10 3 1 0
Time (Years)
G
Patients with N0
Progression-free probability
p = 0.003
Risk
high 103 53 19 9 6 3 1 0
low 122 78 32 20 11 4 2 1
Time (Years)
H
Patients with N1
Progression-free probability
p < 0.0001
Risk
high 110 56 28 10 5 5 2
low 112 68 26 13 6 1 0
Time (Years)
I
Patients with Stage I+II
Progression-free probability
p < 0.0001
Risk
high 142 78 33 14 8 6 3 1
low 189 118 55 32 16 5 2 1
Time (Years)
J
Patients with Stage III+IV
Progression-free probability
p = 0.009
Risk
high 97 45 19 8 4 3 1
low 67 39 10 7 4 1 0
Time (Years)
K
Patients without radiation therapy
Progression-free probability
p = 0.043
Risk
high 82 44 18 7 6 6 3 0
low 96 58 20 9 4 2 2 1
Time (Years)
L
Patients with radiation therapy
Progression-free probability
p < 0.0001
Risk
high 149 76 34 15 6 3 1 1
low 154 99 45 31 17 5 1 0
Time (Years)
