# Supplementary figures and images for: Genomic Instability-Related LncRNA Signature Predicts the Prognosis and Highlights LINC01614 Is a Tumor Microenvironment-Related Oncogenic lncRNA of Papillary Thyroid Carcinoma
Source: Front Oncol. 2021 Sep 16;11:737867. doi: 10.3389/fonc.2021.737867 (PMC8481916; doi:10.3389/fonc.2021.737867)

Group

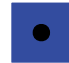

Normal (GTEX+TCGA)

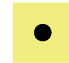

Tumor (TCGA)

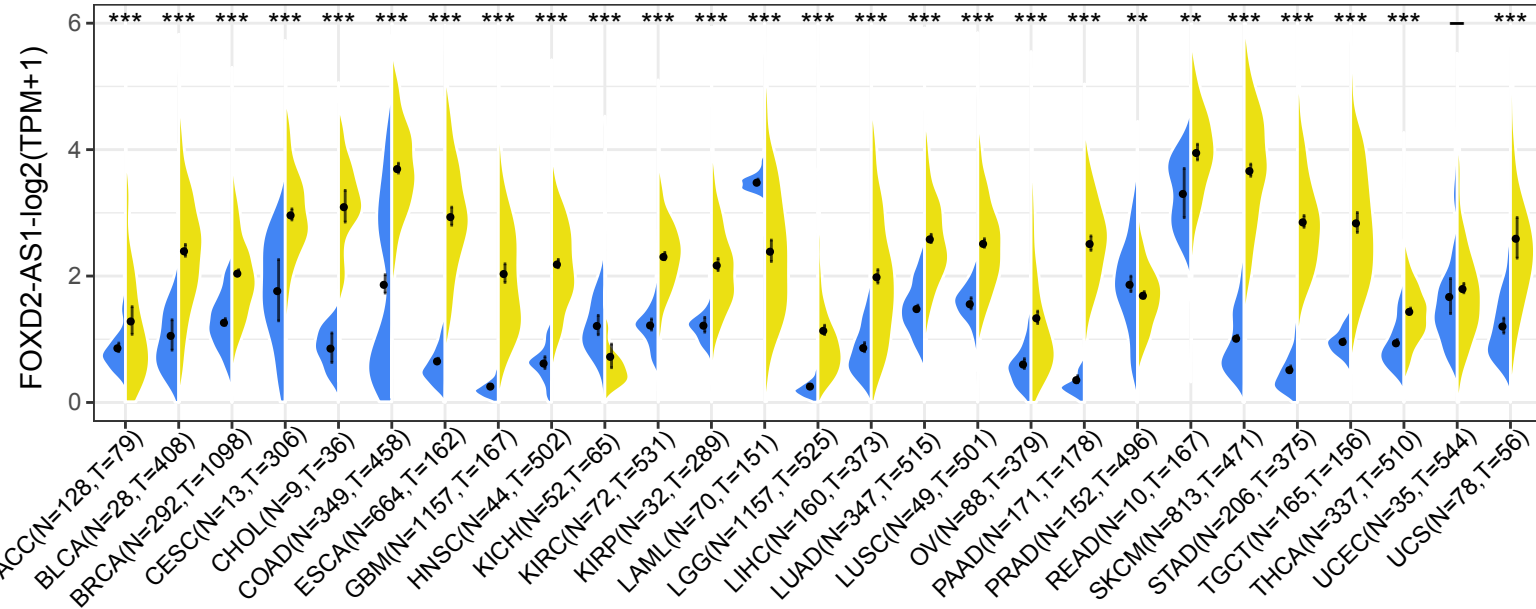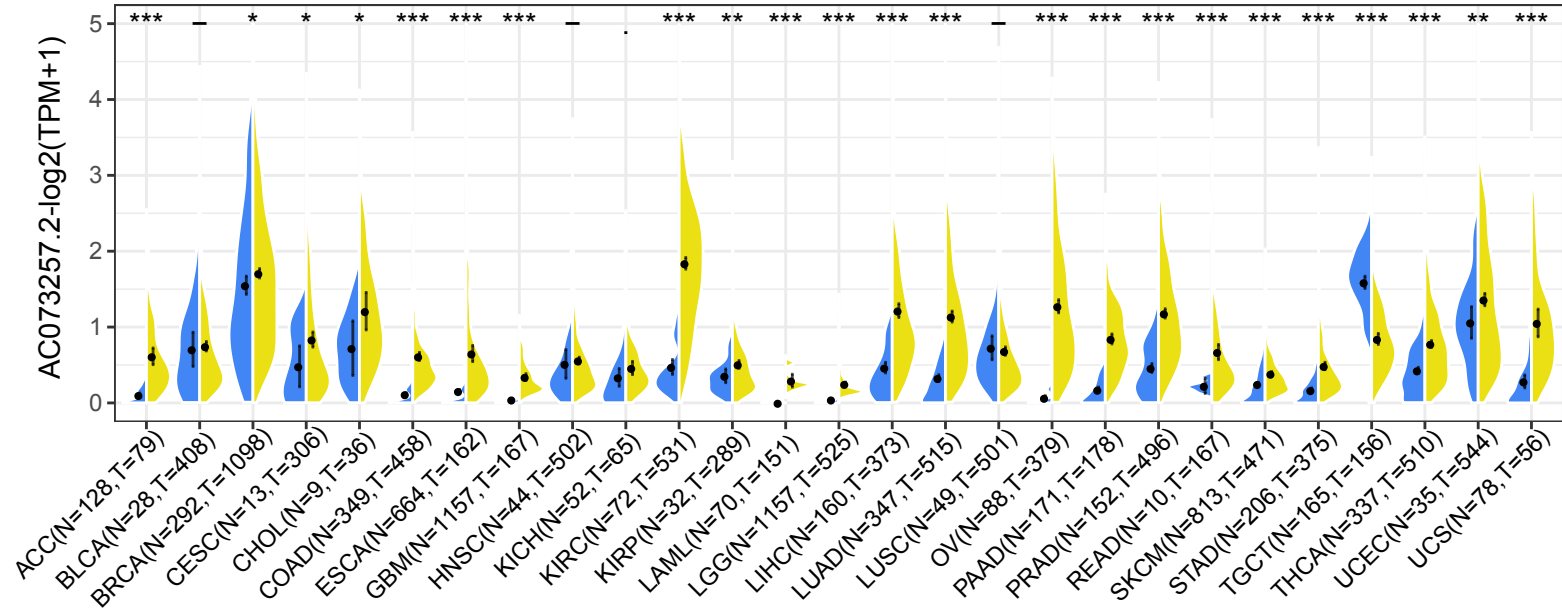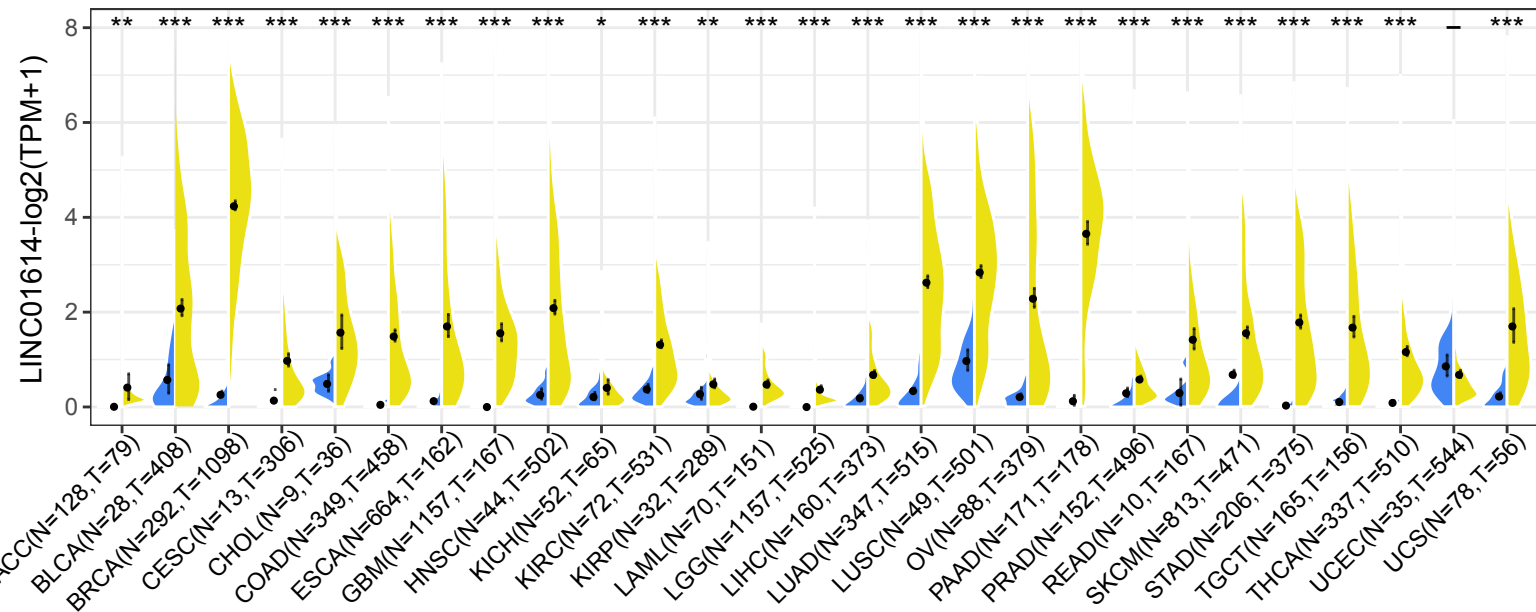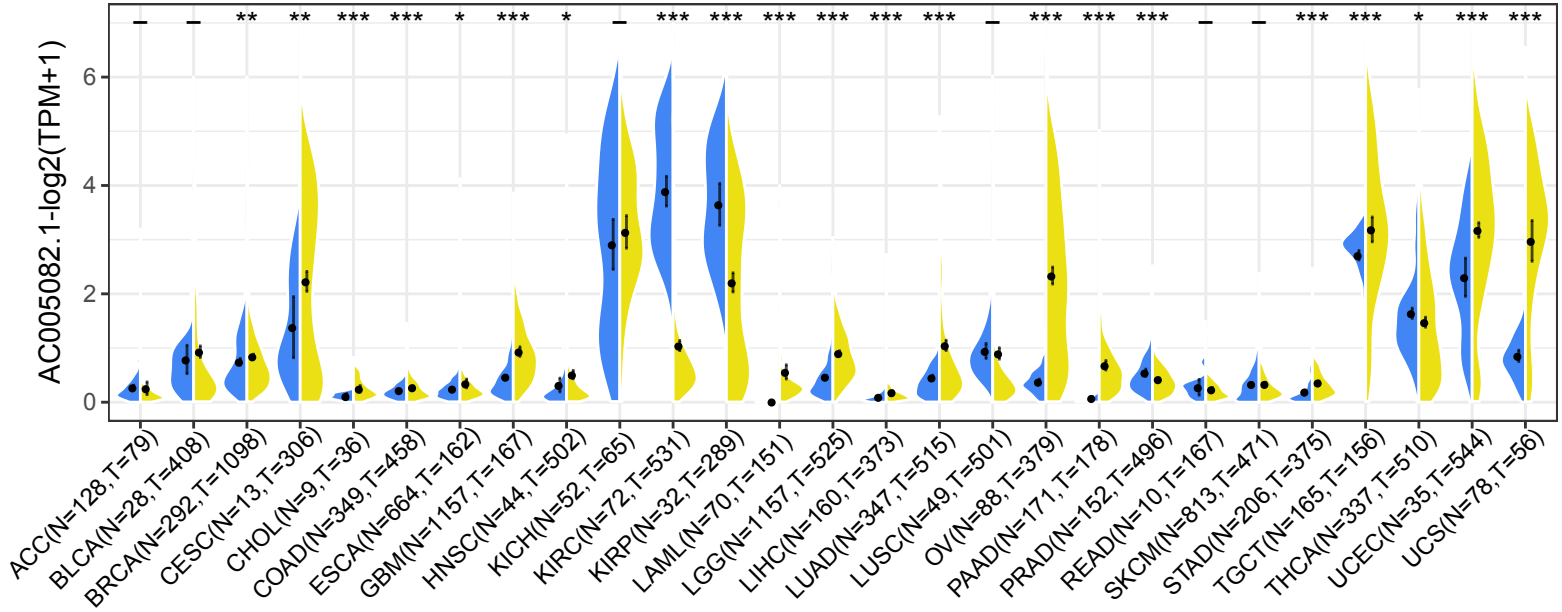

Supplement: Supplementary Figure 2 — The expression pattern of FOXD2-AS1, LINC01614, AC073257.2 and AC005082.1 of pan-cancers in TCGA combined with GTEx. [file Image_2.pdf]
